# Wnt activation protects against neomycin-induced hair cell damage in the mouse cochlea

**DOI:** 10.1038/cddis.2016.35

**Published:** 2016-03-10

**Authors:** L Liu, Y Chen, J Qi, Y Zhang, Y He, W Ni, W Li, S Zhang, S Sun, M M Taketo, L Wang, R Chai, H Li

**Affiliations:** 1 Otorhinolaryngology Department of Affiliated Eye and ENT Hospital, State Key Laboratory of Medical Neurobiology, Fudan University, Shanghai, PR China; 2 Institutes of Biomedical Sciences, Fudan University, Shanghai, PR China; 3 Laboratory Center, Affiliated Eye and ENT Hospital of Fudan University, Shanghai, PR China; 4 Key Laboratory of Hearing Medicine of National Health and Family Planning Commission, Shanghai, PR China; 5 Key Laboratory for Developmental Genes and Human Disease, Ministry of Education, Institute of Life Sciences, Southeast University, Nanjing, China; 6 Co-innovation Center of Neuroregeneration, Nantong University, Nantong, China; 7 Department of Pharmacology, Graduate School of Medicine, Kyoto University, Kyoto, Japan

## Abstract

Recent studies have reported the role of Wnt/*β*-catenin signaling in hair cell (HC) development, regeneration, and differentiation in the mouse cochlea; however, the role of Wnt/*β*-catenin signaling in HC protection remains unknown. In this study, we took advantage of transgenic mice to specifically knockout or overactivate the canonical Wnt signaling mediator *β*-catenin in HCs, which allowed us to investigate the role of Wnt/*β*-catenin signaling in protecting HCs against neomycin-induced damage. We first showed that loss of *β*-catenin in HCs made them more vulnerable to neomycin-induced injury, while constitutive activation of *β*-catenin in HCs reduced HC loss both *in vivo* and *in vitro*. We then showed that loss of *β*-catenin in HCs increased caspase-mediated apoptosis induced by neomycin injury, while *β*-catenin overexpression inhibited caspase-mediated apoptosis. Finally, we demonstrated that loss of *β*-catenin in HCs led to increased expression of forkhead box O3 transcription factor (Foxo3) and Bim along with decreased expression of antioxidant enzymes; thus, there were increased levels of reactive oxygen species (ROS) after neomycin treatment that might be responsible for the increased aminoglycoside sensitivity of HCs. In contrast, *β*-catenin overexpression reduced Foxo3 and Bim expression and ROS levels, suggesting that *β*-catenin is protective against neomycin-induced HC loss. Our findings demonstrate that Wnt/*β*-catenin signaling has an important role in protecting HCs against neomycin-induced HC loss and thus might be a new therapeutic target for the prevention of HC death.

Inner ear hair cells (HCs) are responsible for hearing. Aminoglycosides can be ototoxic and induce caspase-mediated apoptosis in HCs. During mammalian inner ear development, canonical Wnt signaling is critical for otocyst induction and directs the formation of the vestibular organs.^1, 2^ Wnt signaling also has an important role in the cochlear HC development, and knockout of *β*-catenin inhibits HC differentiation from sensory progenitors thus reducing HC generation.^3, 4^ Recently, the Wnt signaling downstream target genes *Lgr5* and *Axin2* have been reported to mark inner ear HC progenitors. Lgr5-positive HC progenitors can self-renew to regenerate HCs after isolation *in vitro* and can spontaneously regenerate HCs after HC damage in the neonatal mouse cochlea *in vivo*.^5, 6, 7, 8, 9, 10^ Recent studies have also shown that Wnt signaling has dual roles in controlling the proliferation and differentiation of HC progenitors;^3, 4^ however, the role of Wnt/*β*-catenin signaling in HC survival and damage protection in the mouse cochlea remains unclear.

In other organs, the Wnt/*β*-catenin signaling pathway has been shown to function in various cell processes, including cellular protection.^11, 12, 13, 14, 15^ The pro-survival activity of the Wnt pathway has been reported in many tissues, and is believed to be mediated by the induction of specific anti-apoptotic genes.^16, 17^ For example, in retinal ganglion cells Wnt activation reduces apoptosis by increasing the expression of protective growth factors including NT3, BDNF, and NGF.^11^ In the intestine, overexpression of Wnt2a glycoprotein ligand of the Wnt proteins decreases bacterial-induced intestinal epithelial cell death. In the liver, Wnt/*β*-catenin signaling acts as a transcriptional co-activator of hypoxia inducible factor-1*α* signaling and has a protective role against hypoxia-induced liver injury. Forkhead box O3 transcription factor (Foxo3) and Bim, which belong to the BCL-2 family members and are the downstream target gene of Foxo3,^18^ have been reported to regulate the expression of stress-response proteins and to be involved in apoptosis in multiple organs.^19, 20, 21^ Overactivation of Wnt signaling inhibits Foxo3-induced apoptosis through upregulation of serum and glucocorticoid-inducible kinase 1 (SGK1),^22^ and overexpression of Wnt/*β*-catenin signaling inhibits Foxo3 signaling in 3,5-diethoxycarbonyl-1,4-dihydrocollidine (DDC)-induced liver injury.^23^ However, the protective role of Wnt/*β*-catenin signaling against neomycin-induced HC loss in the mouse inner ear has been unclear.

In this study, we used loss-of-function and gain-of-function mouse models to investigate the role of Wnt/*β*-catenin signaling in protecting HCs against aminoglycoside-induced ototoxicity in the mouse cochlea both *in vivo* and *in vitro*. We found that *β*-catenin regulates Foxo3 and Bim expression and controls reactive oxygen species (ROS) levels, thus protecting HCs against caspase-mediated apoptosis after neomycin injury.

## Results

### Specifically knockout or overactivate *β*-catenin in HCs

**Figure 1:**
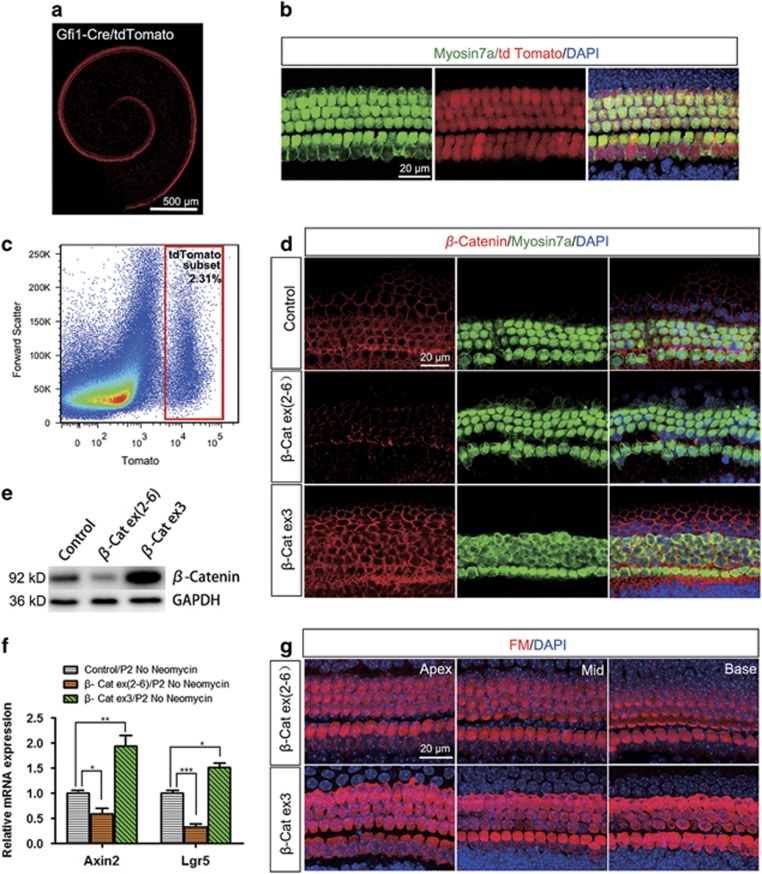
Manipulation of *β*-catenin expression in mouse cochlear HCs. (**a** and **b**) Gfi1-Cre efficiency was tested using Gfi1-Cre/Rosa26-tdTomato mice. Tomato fluorescence showed that Gfi1-Cre activity is similar in inner and outer HCs at all turns in postnatal day (P) 2 Gfi-Cre/tdTomato mice. (**c**) The tdTomato-positive HCs were isolated using flow cytometry. (**d** and **e**) Western blot and immunofluorescence revealed the increased expression of *β*-catenin in Gfi1-Cre/*β*-catenin^flox(exon3)^ mice and decreased expression of *β*-catenin in Gfi1-Cre/*β*-catenin^flox(exon2–6)^ mice. (**f**) qPCR results showed that Wnt target genes, *Axin2* and *Lgr5*, were upregulated in Gfi1-Cre/*β*-catenin^flox(exon3)^ mice and downregulated in Gfi1-Cre/*β*-catenin^flox(exon2–6)^ mice. (**g**) FM1-43 immunofluorescence revealed normal function of mechanotransduction channels in Gfi1-Cre/*β*-catenin^flox(exon3)^ and Gfi1-Cre/*β*-catenin^flox(exon2–6)^ mice. Scale bar: 500 *μ*m (**a**); 20 *μ*m (**b**, **d** and **g**). *P<0.05; **P<0.01; ***P<0.001. *n*=5

*β*-Catenin loss-of-function and gain-of-function experiments were performed using Gfi1-Cre/*β*-catenin^flox(exon2–6)^ and Gfi1-Cre/β-catenin^flox(exon3)^ mice to specifically knockout or overactivate *β*-catenin in HCs. Consistent with a previous report,^24^ we found that Cre is activated in 98.76±0.33% of the HCs and Cre activity is similar among the three turns by using Gfi1-Cre/Rosa26-tdTomato mice (Figures 1a and b). Immunohistochemistry results demonstrated that β-catenin expression in HCs was decreased in Gfi1-Cre/β-catenin^flox(exon2–6)^ mice and increased in Gfi1-Cre/*β*-catenin^flox(exon3)^ mice (Figure 1d). To further test the *β*-catenin knockout or overactivation efficiency in HCs, we generated Gfi1-Cre/*β*-catenin^flox(exon2–6)^/Rosa26-tdTomato and Gfi1-Cre/*β*-catenin^flox(exon3)^/Rosa26-tdTomato mice and isolated the tdTomato-positive HCs using flow cytometry (Figure 1c). Western blot results revealed that the protein expression of *β*-catenin in HCs was significantly decreased in Gfi1-Cre/*β*-catenin^flox(exon2–6)^ mice and increased in Gfi1-Cre/*β*-catenin^flox(exon3)^ mice (Figure 1e). The activation level of Wnt signaling in HCs was further confirmed by the mRNA expression of the Wnt downstream target genes *Axin2* and *Lgr5.* Quantitative real-time PCR (qPCR) results showed that the mRNA expression of *Axin2* and *Lgr5* in HCs were both significantly decreased in Gfi1-Cre/*β*-catenin^flox(exon2–6)^ mice and increased in Gfi1-Cre/*β*-catenin^flox(exon3)^ mice (Figure 1f). Finally, FM1-43, a marker of functional mechanotransduction channels in HCs, was used to detect the mechanotransduction function of HCs in transgenic mice. FM1-43 staining revealed normal function of mechanotransduction channels in HCs in both Gfi1-Cre/*β*-catenin^flox(exon2–6)^ and Gfi1-Cre/*β*-catenin^flox(exon3)^ mice (Figure 1g).

### The Wnt/*β*-catenin pathway was activated in the cochlear HCs after neomycin injury

**Figure 2:**
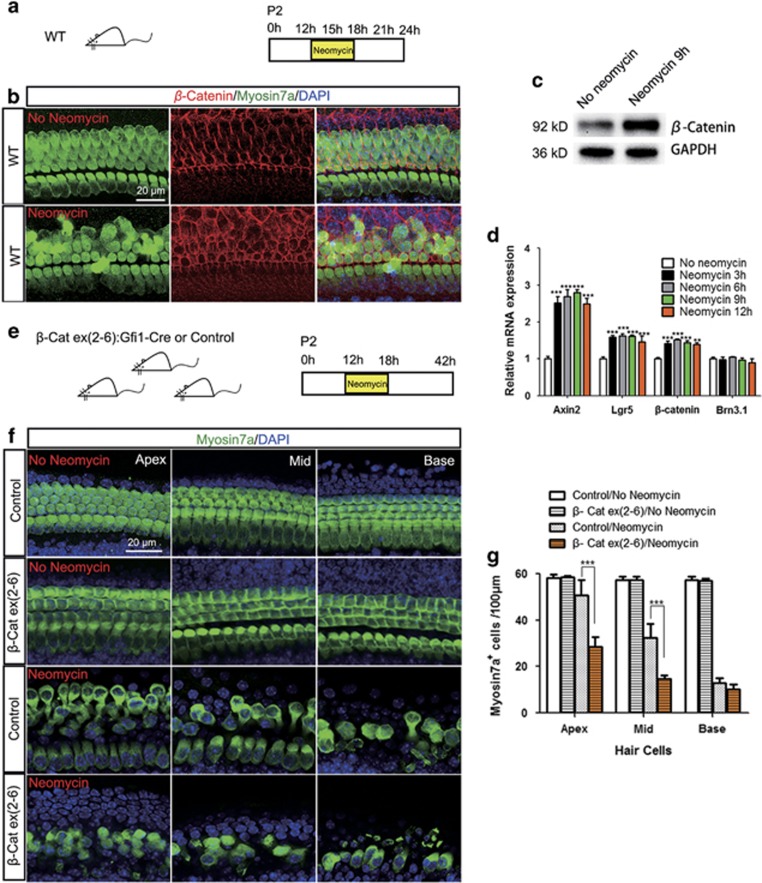
Knockout of *β*-catenin makes HCs more vulnerable to neomycin-induced ototoxicity *in vivo*. (**a**) The diagram of the assay for (**b** and **c**). Cochlear sensory epithelium samples from P2 wild-type mice were dissected out and allowed to recover for 12 h. The samples were treated with 1 mM neomycin for 6 h, allowed to recover for 3 h, and then used for immunostaining and western blot experiments. Samples for qPCR were collected at 0, 3, 6, 9, and 12 h after the beginning of neomycin treatment. (**b** and **c**) Immunofluorescence and western blot revealed the increased *β*-catenin expression in the HCs after neomycin injury. (**d**) qPCR results showed that the expression of *β*-catenin and the Wnt target genes *Axin2* and *Lgr5* were significantly upregulated after neomycin injury. ***P<0.001, **P<0.01, *n*=5, *versus* no neomycin group. (**e**) The diagram of the assay for (**f** and **g**). Cochlear sensory epithelium samples from P2 Gfi1-Cre/*β*-catenin^flox(exon2-6)^ mice were dissected out and allowed to recover for 12 h. The samples were treated with 1 mM neomycin for 6 h, allowed to recover for 24 h, and then stained with Myosin antibody. Littermates lacking the Gfi1-Cre allele were used as controls. (**f** and **g**) *β*-Catenin knockout mice had significantly greater HC loss than control mice in the apical and middle turns of the cochlea after neomycin treatment in newborn mice. Scale bar=20 *μ*m. ***P<0.001, **P<0.01, *n*=5

We next explored the expression of *β*-catenin and Wnt target genes in the cochlear HCs after neomycin treatment. Both immunofluorescence and western blot results revealed increased *β*-catenin expression in the HCs after neomycin injury (Figures 2b and c). qPCR results showed that the expression of *β*-catenin and the Wnt target genes *Axin2* and *Lgr5* were significantly upregulated after neomycin treatment (Figure 2d). These results demonstrated that the Wnt/*β*-catenin pathway was activated in the cochlear HCs after neomycin injury, indicating that Wnt/*β*-catenin might be a protective physiological mechanism against neomycin injury.

### Knockout of *β*-catenin makes the HCs more vulnerable to neomycin-induced ototoxicity *in vitro*

In this experiment, cochlear sensory epithelium samples from postnatal day (P) 2 Gfi1-Cre/*β*-catenin^flox^^(exon2–6)^ and control mice were cultured *in vitro* and treated with neomycin. Without neomycin, there were no reductions in HCs either in control cochleae or in Gfi1-Cre/*β*-catenin^flox(exon2–6)^ cochleae (Figures 2f and g). With neomycin treatment, Gfi1-Cre/*β*-catenin^flox(exon2–6)^ cochleae had significantly greater HC loss than control cochleae in the apical and middle turns (Figures 2f and g and Supplementary Table 1). This result suggested that *β*-catenin has an important role in regulating the sensitivity of cochlear HCs to neomycin-induced injury.

### Overactivation of the Wnt/*β*-catenin signaling pathway protects against neomycin-induced HC damage *in vitro*

**Figure 3:**
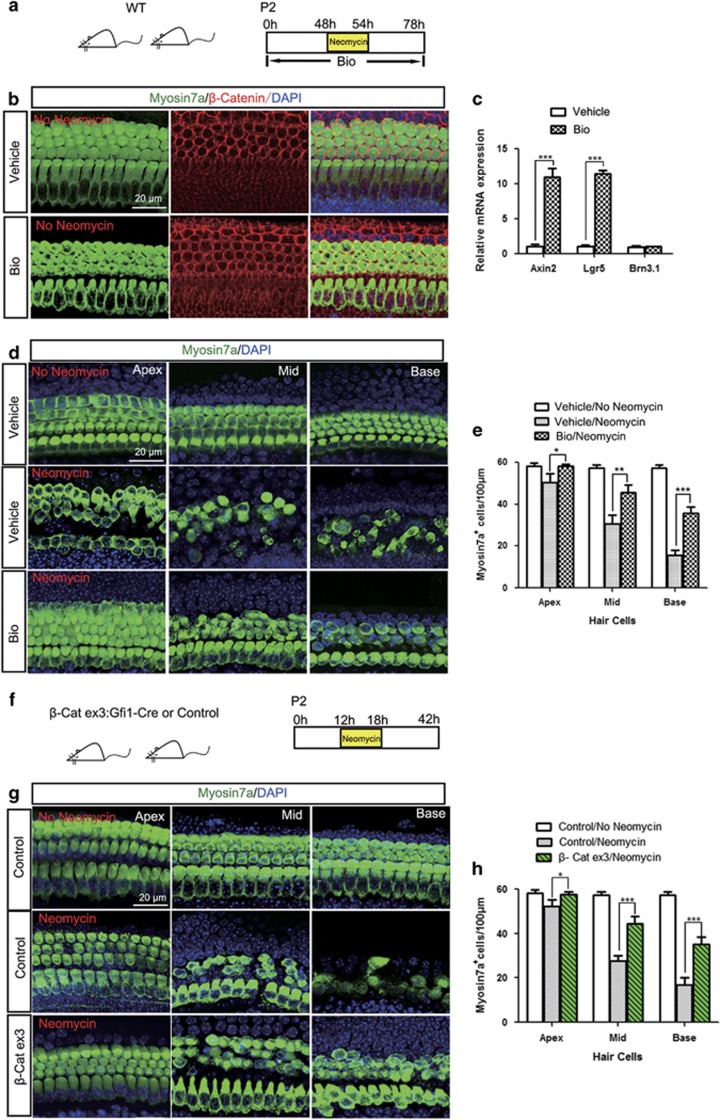
Upregulating the Wnt pathway protects against neomycin-induced HC damage. (**a**) The diagram of the assay for (**d** and **e**). (**b**) P2 cochlear sensory epithelium samples were treated with Bio (5 *μ*M) for 48 h in the absence of neomycin. PBS treatment was the vehicle control. Immunofluorescence photo showed upregulated expression of *β*-catenin in the Bio-treated group (middle turn, HC layer). (**c**) The Wnt target genes *Axin2* and *Lgr5* were upregulated after Bio treatment. (**d**) Bio-treated cochleae had significantly reduced HC loss after neomycin treatment. (**e**) The numbers of Myosin-positive cells in the Bio-treated and control cochleae. (**f**) The diagram of the assay for (**g** and **h**). Cochlear sensory epithelium samples from P2 Gfi1-Cre/*β*-catenin^flox(exon3)^ transgenic mice were dissected out and treated with 1 mM neomycin for 6 h, allowed to recover for 24 h, and then stained with the Myosin7a antibody. Littermates lacking the Gfi1-Cre allele were used as controls. (**g**) Representative photo of Myosin-positive HCs after neomycin treatment in controls and Gfi1-Cre/*β*-catenin^flox(exon3)^ mice. (**h**) Statistical data showing that Gfi1-Cre/*β*-catenin^flox(exon3)^ transgenic mice had more Myosin7a-positive HCs in the apical, middle, and basal turns of the cochlear epithelium. Scale bar=20 *μ*m. *P<0.05; **P<0.01; ***P<0.001. *n*=5

To investigate the protective role of *β*-catenin against neomycin-induced HC damage, we used the Wnt agonist Bio and *β*-catenin overexpressing transgenic mice in two independent *in vitro* experiments. First, cochlear sensory epitheliums from P2 wild-type (WT) mice were cultured with Bio (5 *μ*M) for 48 h (Figure 3a). Immunofluorescence results demonstrated upregulated expression of *β*-catenin in Bio-treated HCs (Figure 3b). qPCR data showed that Bio-treated cochleae had significantly higher expression of the Wnt downstream target genes *Lgr5* and *Axin2* than the control group (Figure 3c). When treated with neomycin in the presence of Bio, Myosin7a staining showed that Bio-treated cochleae had significantly less HC loss than the control cochleae in all three turns (Figures 3d and e and Supplementary Table 1), which suggested that Wnt/*β*-catenin signaling could protect HCs against neomycin-induced damage *in vitro.*

In a separate experiment, we investigated the protective role of *β*-catenin using Gfi1-Cre/*β*-catenin^flox(exon3)^ transgenic mice. Compared with control cochleae, Gfi1-Cre/*β*-catenin^flox(exon3)^ cochleae had significantly reduced HC loss in all three turns after neomycin treatment (Figures 3g and h and Supplementary Table 1), which was consistent with the Wnt agonist treatment and demonstrated that overexpression of Wnt/*β*-catenin protects against neomycin-induced HC damage *in vitro.*

### Knockout of *β*-catenin in HCs leads to partial hearing loss and scattered HC loss *in vivo*

**Figure 4:**
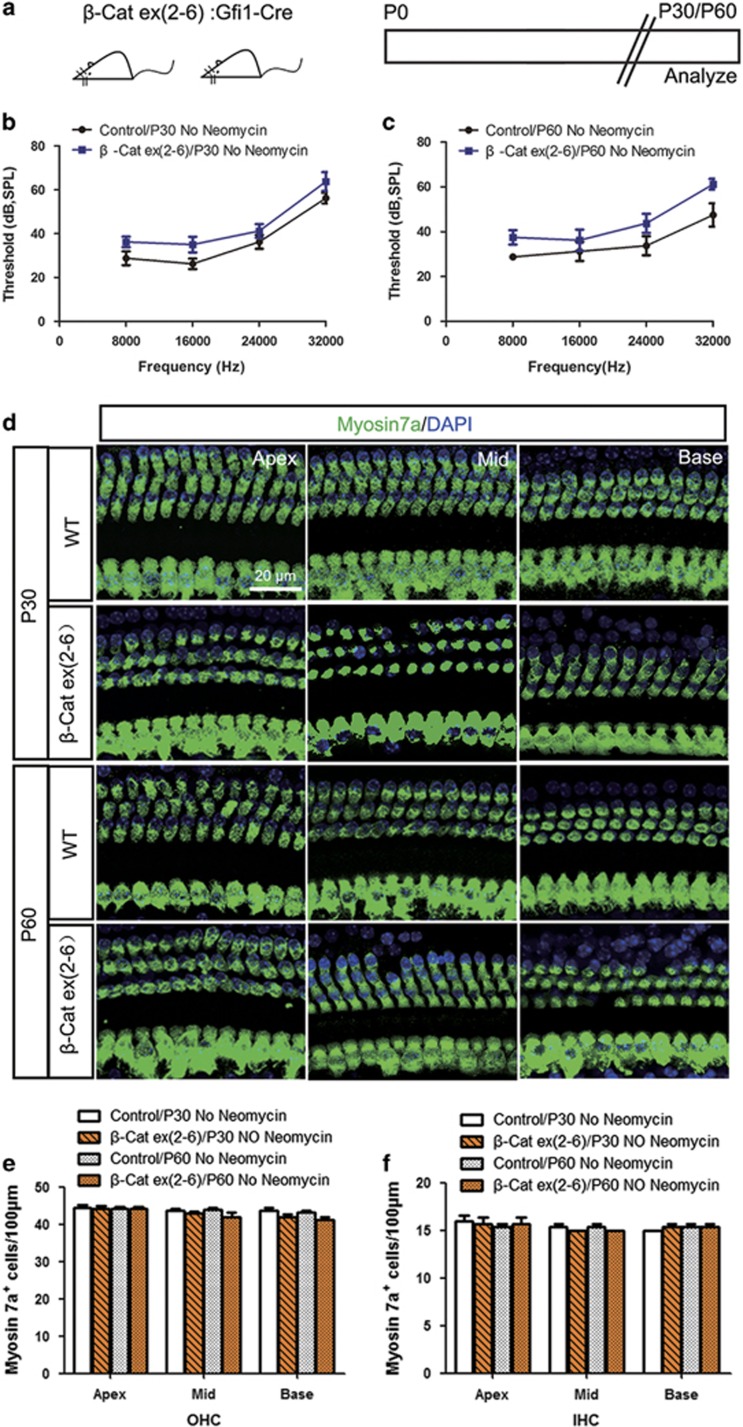
Knockout of *β*-catenin in HCs leads to partial hearing loss and scattered HC loss *in vivo*. (**a**) The scheme of the assay. Gfi1-Cre/*β*-catenin^flox(exon2–6)^ transgenic mice were analyzed at P30 and P60 without neomycin treatment. (**b** and **c**) Pure-tone ABR data showed partial hearing loss at P30 and P60 in Gfi1-Cre/*β*-catenin^flox(exon2–6)^ mice compared with control groups. (**d**) Myosin7a immunofluorescence showed scattered HC loss in the middle and basal turns in Gfi1-Cre/*β*-catenin^flox(exon2-6)^ transgenic mice. (**e** and **f**) Statistical data showing that the total HC number was not significantly different between Gfi1-Cre/*β*-catenin^flox(exon2–6)^ and control mice. Scale bar=20 *μ*m. *n*=5

To investigate the role of *β*-catenin in HC survival *in vivo*, we measured hearing function of Gfi1-Cre/*β*-catenin^flox(exon2–6)^ mice using pure-tone auditory brainstem response (ABR) and then dissected out the cochlear sensory epithelium for immunohistochemistry staining at P30 and P60 (Figure 4a). The same litter control mice have normal hearing, and no HC loss was observed at P30 or P60 (Figures 4b and d). In Gfi1-Cre/*β*-catenin^flox(exon2–6)^ mice, we observed a 5–10 dB threshold shift at P30 and a 5–15 dB threshold shift at P60 compared with controls (Figures 4c and d). We also found scattered HC loss in middle and basal turns, but the total HC number showed no significant difference compared with controls at P30 and P60 (Figures 4e and f). These results demonstrated that deletion of *β*-catenin in HCs leads to partial hearing loss and scattered HC loss *in vivo.*

### Overexpression of Wnt/*β*-catenin protects against neomycin-induced hearing loss and HC loss *in vivo*

**Figure 5:**
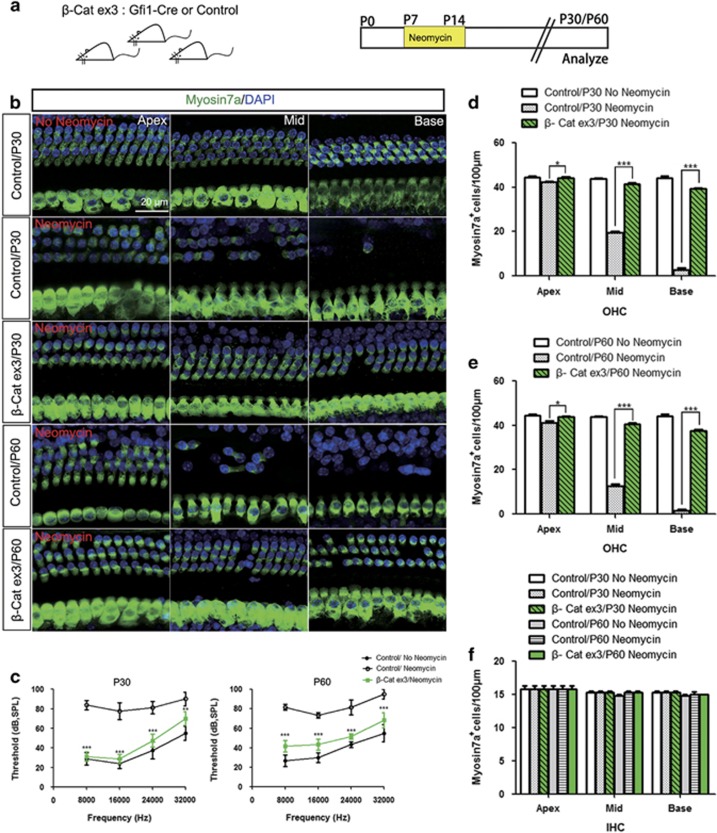
Overexpression of Wnt/*β*-catenin protects against neomycin-induced hearing loss and HC loss *in vivo*. (**a**) The scheme of the assay. Gfi1-Cre/*β*-catenin^flox(exon3)^ transgenic mice were given daily subcutaneous injections of neomycin (200 mg/kg) from P7 to P14. At P30 and P60, hearing function was measured by using pure-tone ABR and then the cochlear sensory epithelium was dissected out for immunohistochemistry staining. (**b**) Myosin7a immunofluorescence in P30 and P60 mice after the injection of neomycin or saline. (**c**) ABR data in P30 and P60 mice. (**d**) The numbers of Myosin-positive OHC in P30 mice. (**e**) The numbers of Myosin-positive OHC in P60 mice. (**f**) The numbers of Myosin-positive IHC in P30 and P60 mice. Scale bar=20 *μ*m. *P<0.05; ***P<0.001. *n*=5

Here, Gfi1-Cre/*β*-catenin^flox(exon3)^ transgenic mice were given daily subcutaneous injections of neomycin from P7 to P14, which is the ototoxic-sensitive period in the cochlea.^25^ At P30 and P60, we measured hearing function and then dissected out the cochlear sensory epithelium for immunohistochemistry staining (Figure 5a). Control mice had significant hearing loss, and the ABR thresholds were significantly increased at all frequencies at both P30 and P60 (Figure 5c). In Gfi1-Cre/*β*-catenin^flox(exon3)^ mice, the ABR threshold shifts were significantly lower at all frequencies compared with the control littermates at both P30 and P60 (Figure 5c), suggesting that overexpression of Wnt/*β*-catenin protects against neomycin-induced hearing loss *in vivo.* Immunohistochemistry results showed that Gfi1-Cre/*β*-catenin^flox(exon3)^ mice had significantly reduced outer hair cell (OHC) loss compared with the control littermates at both P30 and P60 (Figures 5b, d and e and Supplementary Table 2). This demonstrated that overexpression of Wnt/*β*-catenin protects against neomycin-induced OHC loss *in vivo.* There was almost no inner hair cell (IHC) loss in either Gfi1-Cre/*β*-catenin^flox(exon3)^ mice or control littermates at P30 or P60 (Figures 5b and f).

### Wnt/*β*-catenin regulates the caspase-mediated apoptosis induced by neomycin injection *in vivo*

**Figure 6:**
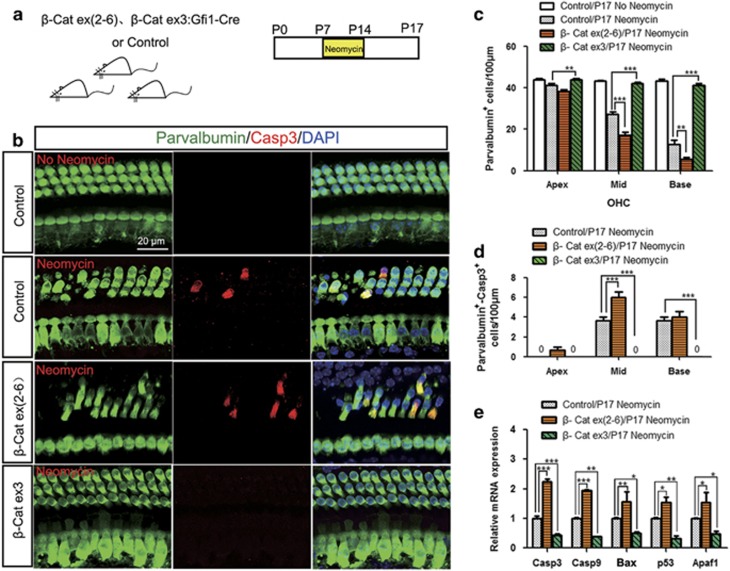
Wnt/*β*-catenin regulates the caspase-mediated apoptosis that is induced by neomycin injection *in vivo*. (**a**) The scheme of the assay. Mice were given daily subcutaneous injections of neomycin (200 mg/kg) from P7 to P14. At P17, cochlear sensory epithelium samples were dissected out. The age-matched littermates lacking the Gfi1-Cre allele were used as controls. (**b**) Representative confocal images of the HC marker parvalbumin and the apoptosis marker cleaved caspase-3 (Casp3) immunofluorescence in P17 mice (middle turn, HC layer). (**c**) Parvalbumin-positive HC quantification in OHCs in P17 mice. (**d**) Statistical data showing that Parvalbumin/Casp3 double-positive cells significantly increased in Gfi1-Cre/*β*-catenin^flox(exon2–6)^ mice and decreased in Gfi1-Cre/*β*-catenin^flox(exon3)^ mice. (**e**) qPCR analysis showed that Casp3, Casp9, Bax, p53, and Apaf1 expression increased in Gfi1-Cre/*β*-catenin^flox(exon2–6)^ mice and decreased in Gfi1-Cre/*β*-catenin^flox(exon3)^ mice after neomycin damage. Scale bar=20 *μ*m. *P<0.05; **P<0.01; ***P<0.001. *n*=5 (**e**) or 8 (**b**–**d**)

Previous studies reported that neomycin kills HCs through the induction of apoptosis;^25, 26, 27^ thus, we investigated the expression of apoptosis-related genes after neomycin injury *in vivo*. Gfi1-Cre/*β*-catenin^flox(exon3)^ and Gfi1-Cre/*β*-catenin^flox(exon2–6)^ mice were treated with neomycin and were killed 3 days after the last injection (Figure 6a). Immunohistochemistry data showed that Gfi1-Cre/*β*-catenin^flox(exon3)^ cochleae had no parvalbumin/caspase-3 double-positive cells in all three turns, while Gfi1-Cre/*β*-catenin^flox(exon2–6)^ cochleae had significantly more parvalbumin/caspase-3 double-positive cells in the middle turn compared with the control littermates (Figures 6b and d and Supplementary Table 3). qPCR data showed that Gfi1-Cre/*β*-catenin^flox(exon3)^ mice had significantly lower expression of the pro-apoptotic genes *Casp3, Casp9, Bax, p53*, and *Apaf1* (Figure 6e), while Gfi1-Cre/*β*-catenin^flox(exon2–6)^ mice had significantly higher expression of pro-apoptotic genes (Figure 6e). These results demonstrated that Wnt/*β*-catenin signaling could regulate the caspase-mediated apoptosis induced by neomycin injection *in vivo*.

### Caspase-mediated HC apoptosis is regulated by Wnt/*β*-catenin signaling in HCs after neomycin injury *in vitro*

**Figure 7:**
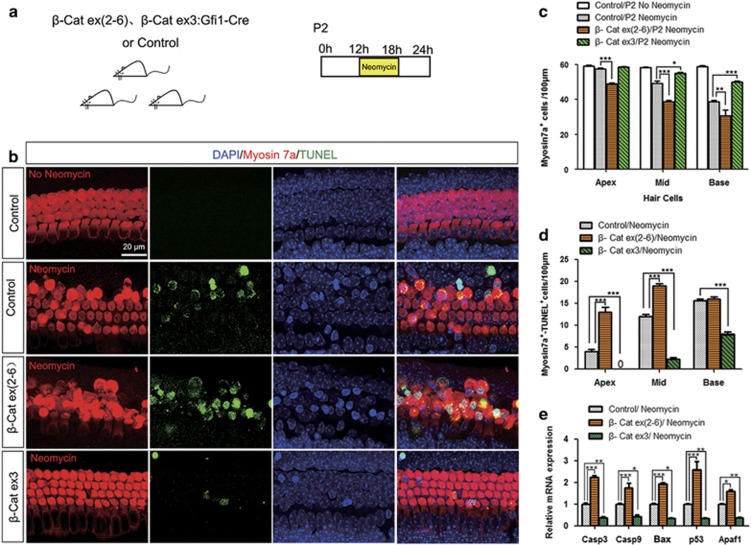
Caspase-mediated HC apoptosis is regulated by Wnt/*β*-catenin signaling in HCs after neomycin injury *in vitro*. (**a**) The scheme of the assay. Cochlear sensory epithelium samples were dissected out from P2 Gfi1-Cre/*β*-catenin^flox(exon3)^ and Gfi1-Cre/*β*-catenin^flox(exon2–6)^ mice, then cultured with 1 mM neomycin for 6 h and allowed to recover for 6 h before analysis. (**b**) Representative confocal images of TUNEL and Myosin immunofluorescence (middle turn, HC layer). (**c**) Numbers of Myosin-positive HCs after neomycin treatment. (**d**) Statistical data showing that TUNEL/Myosin double-positive cells significantly increased in Gfi1-Cre/*β*-catenin^flox(exon2–6)^ mice and decreased in Gfi1-Cre/*β*-catenin^flox(exon3)^ mice. (**e**) qPCR analysis showing that Casp3, Casp9, Bax, p53, and Apaf1 expression increased in Gfi1-Cre/*β*-catenin^flox(exon2–6)^ mice and decreased in Gfi1-Cre/*β*-catenin^flox(exon3)^ mice after neomycin damage. Scale bar=20 *μ*m. *P<0.05; **P<0.01; ***P<0.001. *n*=5

After neomycin treatment *in vitro*, Gfi1-Cre/*β*-catenin^flox(exon3)^ cochleae had significantly fewer TUNEL/Myosin7a double-positive cells compared with the control littermates, while Gfi1-Cre/*β*-catenin^flox^^(exon2–6)^ cochleae had significantly more TUNEL/Myosin7a double-positive cells in the apical and middle turns (Figures 7b and d and Supplementary Table 4). qPCR data showed that the expression of pro-apoptotic genes *Casp3, Casp9, Bax, p53*, and *Apaf1* was significantly reduced in Gfi1-Cre/*β*-catenin^flox(exon3)^ cochleae compared with controls and was significantly increased in Gfi1-Cre/*β*-catenin^flox(exon2–6)^ cochleae (Figure 7e). These results demonstrated that Wnt/*β*-catenin signaling in HCs inhibits the caspase-mediated apoptosis induced by neomycin *in vitro*.

### Foxo3 expression is regulated by Wnt/*β*-catenin signaling in HCs after neomycin injury

**Figure 8:**
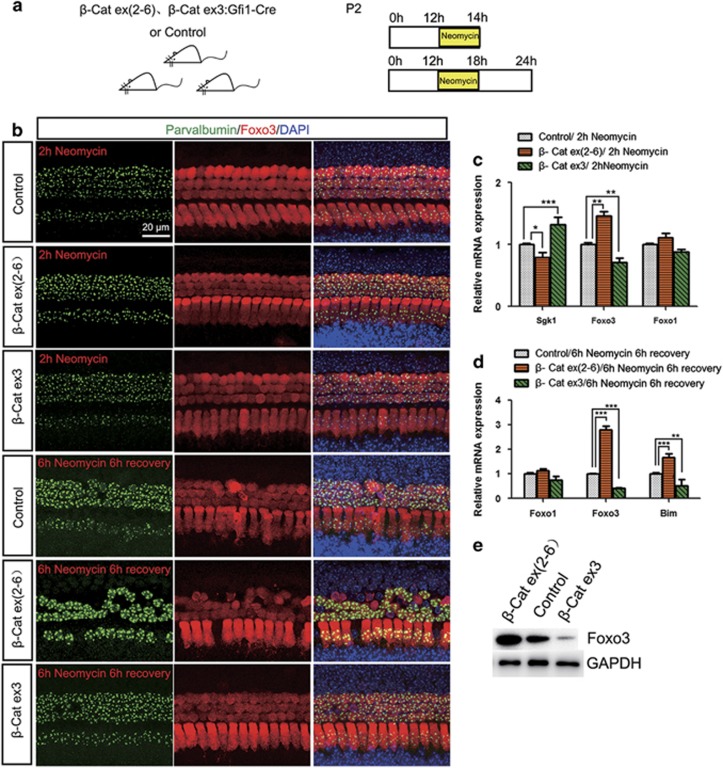
Foxo3 expression is regulated by Wnt/*β*-catenin signaling in HCs after neomycin injury. (**a**) The scheme of the assay. In the first experiment, P2 mouse cochlear epithelium samples were cultured with neomycin for 2 h. In the second experiment, P2 mouse cochlear epithelium samples were cultured with neomycin for 6 h and then allowed to recover for 6 h before analysis. (**b**) Parvalbumin and Foxo3 immunofluorescence showed that Foxo3 expression increased in Gfi1-Cre/*β*-catenin^flox(exon2–6)^ mice and decreased in Gfi1-Cre/*β*-catenin^flox(exon3)^ mice after neomycin damage (middle turn, HC layer). (**c** and **d**) qPCR data showed the mRNA expression level of *Sgk1*, *Foxo3*, *Foxo1*, and *Bim* after neomycin treatment. (**e**) Western blot showed that Foxo3 expression increased in Gfi1-Cre/*β*-catenin^flox(exon2–6)^ mice and decreased in Gfi1-Cre/*β*-catenin^flox(exon3)^ mice after neomycin damage. Scale bar=20 *μ*m. *P<0.05; **P<0.01; ***P<0.001. *n*=5 (**b**, **c**, and **e**) or 8 (**d**)

Previous studies have reported that Wnt/*β*-catenin inhibits the pro-apoptotic transcription factor Foxo3 and protects against oxidative stress-induced apoptosis though downregulation of Foxo3.^14, 15, 28^ We investigated the Foxo3 expression in neomycin-treated cochleae. At 6 h after neomycin treatment, intense nuclear Foxo3 staining was observed in control HCs, which is indicative of active Foxo3 signaling in response to neomycin-induced HC damage (Figure 8b). In Gfi1-Cre/*β*-catenin^flox(exon3)^ cochleae, HCs had significantly reduced Foxo3 staining intensity (Figure 8b). In Gfi1-Cre/*β*-catenin^flox(exon2–6)^ cochleae, HCs had significantly greater Foxo3 staining intensity (Figure 8b). qPCR and western blot data showed that Foxo3 expression was significantly reduced in Gfi1-Cre/*β*-catenin^flox(exon3)^ cochleae and significantly increased in Gfi1-Cre/*β*-catenin^flox(exon2–6)^ cochleae compared with the controls (Figures 8d and e). Bim is one of the BCL-2 family members participating in the progress of apoptosis, and is the downstream target gene of Foxo3.^18^ qPCR data revealed that Bim expression was also significantly decreased in Gfi1-Cre/*β*-catenin^flox(exon3)^ cochleae and increased in Gfi1-Cre/*β*-catenin^flox(exon2–6)^ cochleae (Figure 8d). These results suggested that after neomycin injury Foxo3 and Bim expression was inhibited when *β*-catenin was overexpressed in HCs and increased when *β*-catenin was knocked out in HCs. We also noticed that the mRNA expression of Foxo1, which is another member of the Foxo protein superfamily, was not significantly changed (Figure 8d), suggesting that Foxo1 might not be regulated by Wnt signaling in the cochlea.

Furthermore, we investigated the expression level of Foxo3 after 2 h neomycin treatment, at which time no obvious HC loss has occurred yet. Immunohistochemistry results showed that HCs had significantly reduced Foxo3 expression in Gfi1-Cre/*β*-catenin^flox(exon3)^ cochleae and had significantly higher Foxo3 expression in Gfi1-Cre/*β*-catenin^flox(exon2–6)^ cochleae (Figure 8b). qPCR data also demonstrated that the expression of Foxo3 was significantly decreased in Gfi1-Cre/*β*-catenin^flox(exon3)^ cochleae and significantly increased in Gfi1-Cre/*β*-catenin^flox(exon2–6)^ cochleae (Figure 8c). The expression of Sgk1, which is a *β*-catenin target gene and the upstream inhibitor of Foxo3 activity,^23^ was significantly increased in Gfi1-Cre/*β*-catenin^flox(exon3)^ cochleae and significantly decreased in Gfi1-Cre/*β*-catenin^flox(exon2–6)^ cochleae (Figure 8c). Altogether, these results suggested that Foxo3 expression in HCs is regulated by Wnt/*β*-catenin signaling after neomycin injury.

### ROS levels in HCs are regulated by Wnt/*β*-catenin signaling after neomycin injury

**Figure 9:**
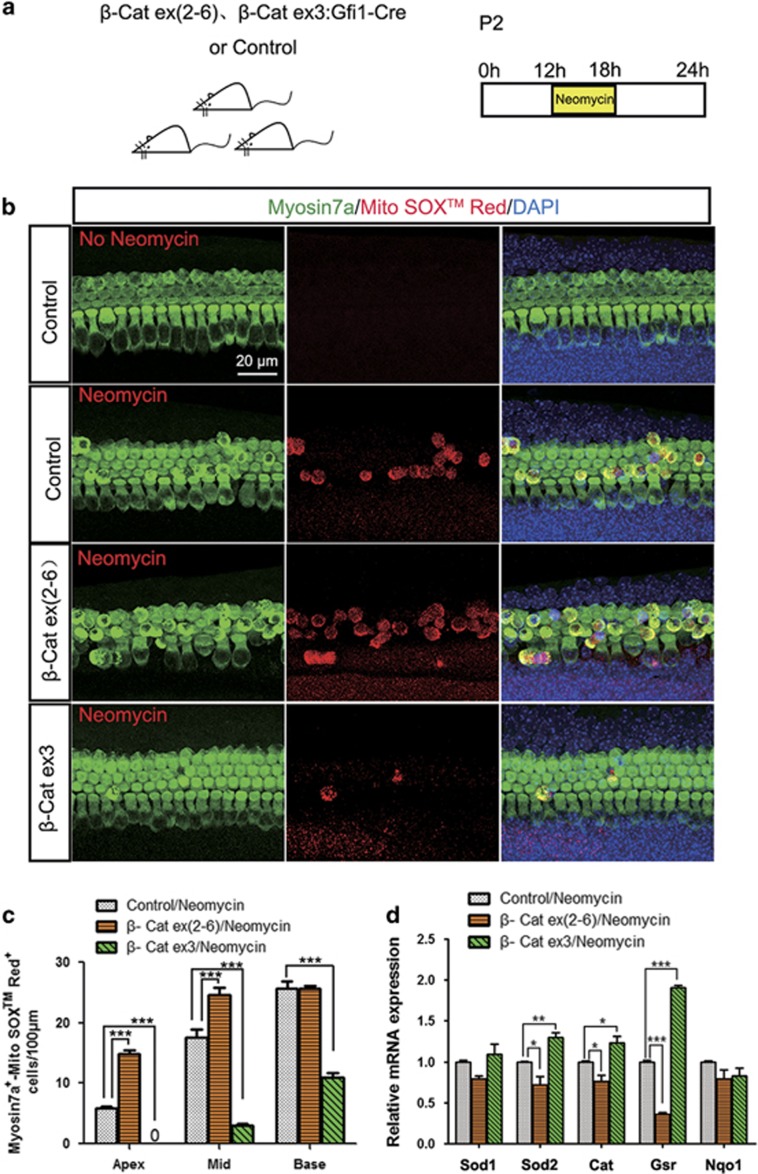
ROS levels in HCs are regulated by Wnt/*β*-catenin signaling after neomycin injury. (**a**) The scheme of the assay. Cochlear epithelium samples were dissected out and cultured with 1 mM neomycin for 6 h and allowed to recover for 6 h before analysis. (**b**) Myosin7a and MitoSOX Red immunofluorescence showed that ROS levels increased in Gfi1-Cre/*β*-catenin^flox(Ex2–6)^ mice and decreased in Gfi1-Cre/*β*-catenin^flox(exon3)^ mice after neomycin damage (middle turn, HC layer). (**c**) The numbers of Myosin7a/MitoSOX Red double-positive cells. (**d**) qPCR results showed that antioxidant gene expression significantly decreased Gfi1-Cre/*β*-catenin^flox(exon2–6)^ mice but significantly increased in Gfi1-Cre/*β*-catenin^flox(exon3)^ mice. Scale bar=20 *μ*m. *P<0.05; **P<0.01; ***P<0.001. *n*=5

Previous studies reported that Foxo3 is required for the regulation of oxidative stress,^18, 29^ and Wnt/*β*-catenin has been reported to protect against oxidative stress-induced apoptosis in many organs.^14, 15^ In the mouse cochlea, aminoglycoside-induced accumulation of ROS is closely related to HC apoptosis.^30^ In this experiment, we used MitoSOX Red, a redox fluorophore that selectively detects mitochondrial superoxide,^31, 32, 33^ to evaluate mitochondrial ROS generation in HCs after neomycin treatment (Figure 9a). Results showed that Gfi1-Cre/*β*-catenin^flox(exon3)^ cochleae had significantly fewer MitoSOX/Myosin7a double-positive cells in all three turns compared with control, and Gfi1-Cre/*β*-catenin^flox(exon2–6)^ cochleae had significantly more MitoSOX/Myosin7a double-positive cells in the apical turn (Figures 9b and c and Supplementary Table 5). These results suggested that mitochondrial ROS levels in HCs are significantly reduced in Gfi1-Cre/*β*-catenin^flox(exon3)^ mice and significantly increased in Gfi1-Cre/*β*-catenin^flox(exon2–6)^ mice. To find out how Wnt/*β*-catenin regulates ROS levels in *β*-catenin overexpressed/knockout HCs, qPCR was used to investigate the expression levels of several antioxidant enzymes (Nqo1, Cat, Sod1, Sod2, and Gsr). Results showed that antioxidant genes expression, including Sod2, Cat, and Gsr, were significantly increased in Gfi1-Cre/*β*-catenin^flox(exon2–6)^ mice but significantly decreased in Gfi1-Cre/*β*-catenin^flox(exon3)^ mice (Figure 9d). All of these results demonstrated that Wnt/*β*-catenin signaling regulates neomycin-induced ROS accumulation in HCs.

### Antioxidant treatment rescues *β*-catenin deficiency-induced HC loss after neomycin injury

**Figure 10:**
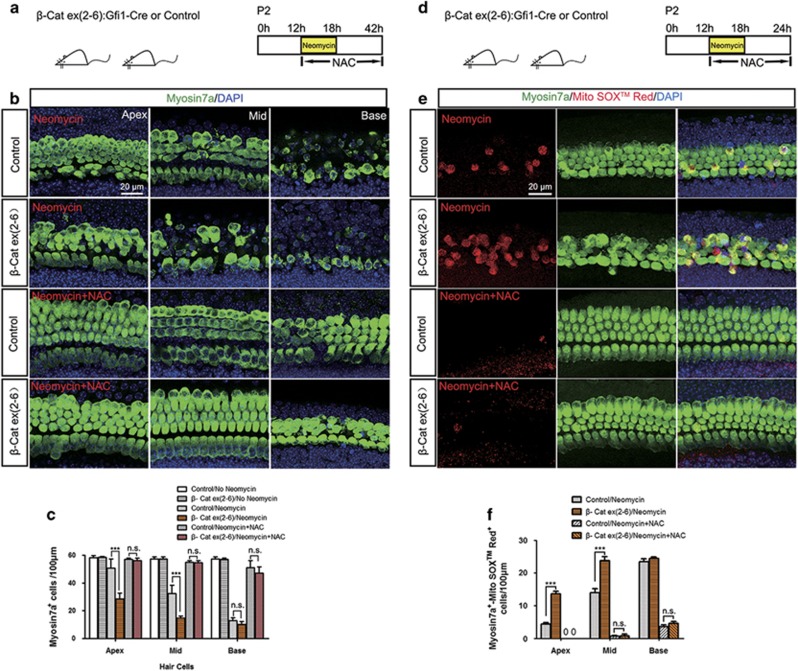
The NAC rescue assay. (**a**) The scheme of the assay for (**b** and **c**). P2 Gfi1-Cre/*β*-catenin^flox(exon2–6)^ cochlear epithelium samples were dissected out and cultured with 1 mM neomycin for 6 h with NAC (20 *μ*M), then allowed to recover for 24 h in the presence of NAC before analysis. (**b**) Myosin7a immunofluorescence showed that NAC treatment rescued *β*-catenin-deficient HCs from neomycin injury. (**c**) Statistical data showing that the number of surviving HCs was not significantly different between Gfi1-Cre/*β*-catenin^flox(exon2–6)^ and control mice after NAC treatment. (**d**) The scheme of the assay for (**e** and **f)**. P2 Gfi1-Cre/*β*-catenin^flox(exon2–6)^ cochlear epithelium samples were dissected out and cultured with 1 mM neomycin for 6 h with NAC (20 *μ*M), then allowed to recover for 6 h in the presence of NAC before analysis. (**e**) MitoSOX Red immunofluorescence showed that ROS levels significantly decreased in the HCs of Gfi1-Cre/*β*-catenin^flox(exon2–6)^ mice after NAC treatment. (**f**) The number of Myosin7a/MitoSOX Red double-positive cells. Scale bar=20 *μ*m. ***P<0.001. n.s., no significant difference. *n*=5

To further investigate whether the increase in ROS levels contributes to the increased injury sensitivity to aminoglycosides of *β*-catenin-deficient HCs, the antioxidant *N*-acetylcysteine (NAC), which is a reduced glutathione provider and a direct scavenger of reactive oxygen intermediates,^34^ was used to treat the explant cultured cochlea with neomycin injury. After NAC treatment, HC loss dramatically decreased in both Gfi1-Cre/β-catenin^flox(exon2–6)^ and control mice, and the number of surviving HCs was not significantly different between Gfi1-Cre/*β*-catenin^flox(exon2–6)^ and control mice (Figures 10b and c). Moreover, MitoSOX Red immunofluorescence showed that ROS levels significantly decreased in HCs of Gfi1-Cre/*β*-catenin^flox(exon2–6)^ mice after NAC treatment (Figures 10e and f), suggesting that the rescue of the HCs was associated with a decrease in oxidative stress. Together, these results showed that antioxidant treatment successfully rescued the *β*-catenin deficiency-induced HC loss in Gfi1-Cre/*β*-catenin^flox(exon2–6)^ mice after neomycin injury, and demonstrated that ROS accumulation was the major cause of the high injury sensitivity to aminoglycosides in *β*-catenin-deficient HCs.

## Discussion

The role of Wnt/*β*-catenin signaling in cochlear development and HC regeneration has been extensively studied in the mouse inner ear. Recently, Wnt/*β*-catenin has been reported to be required for HC differentiation in the mouse cochlea. Knockout of *β*-catenin inhibits prosensory cells from differentiating into HCs, but *β*-catenin is not required to maintain HC fate once it is specified.^4^ However, whether Wnt/*β*-catenin signaling is required for HC survival has not been investigated. In this study, we observed increased susceptibility of HCs to neomycin treatment in Gfi1-Cre/*β*-catenin^flox(exon2–6)^ mice, which indicated that *β*-catenin might have an important role in protecting the sensory HCs.

Previous studies reported that several pathways are involved in *β*-catenin deficiency-induced cell death, including increased apoptosis of hepatic progenitor cells due to enhanced expression of cleaved caspase-9 and caspase-3 when *β*-catenin expression is blocked,^16^ increased apoptosis in cisplatin-resistant lung adenocarcinoma cells when DKK3 is used to inhibit the Wnt/*β*-catenin pathway,^35^ and increased si-LGR5-induced apoptosis when the mitochondrial membrane potential is disrupted in colorectal cancer cells.^36^ In our study, significantly greater HC loss was observed in *β*-catenin knockout cochleae compared with controls after neomycin treatment (Figure 2), which was accompanied by upregulation of the pro-apoptotic transcription factor Foxo3 and its downstream target gene Bim (Figure 8). Foxo3 is a pro-apoptotic transcription factor that regulates the expression of stress-response proteins and leads to apoptosis in many tissues.^19, 20, 21^ In neuronal cells, activation of Foxo3 induces two sequential ROS waves by induction of its transcriptional target Bim.^18^ ROS can oxidize cell constituents, such as DNA, and can lead to DNA damage that activates multiple apoptotic pathways, including caspase-mediated apoptosis and p53-dependent apoptosis.^37, 38, 39^ Foxo3 also activates an ROS rescue pathway by inducing Sestrin3, which is responsible for the biphasic ROS accumulation.^18^ Previous studies have reported that Wnt/*β*-catenin protects against oxidative stress-induced apoptosis through downregulation of Foxo3.^14, 15, 28^ In *β*-catenin-deficient cochleae, we found that upregulation of Foxo3 expression was accompanied by decreased expression of antioxidant enzymes (Figure 9), increased mitochondrial ROS accumulation (Figure 9), and significantly higher expression levels of Casp3, Casp9, Bax, Apaf1, and p53 (Figures 6 and 7), suggesting that the increased susceptibility of *β*-catenin-deficient HCs to neomycin treatment is attributed to Foxo3 activation and ROS accumulation. The precise role of Foxo3 in the oxidative stress in cochlear HCs needs to be investigated in the future.

Previous studies reported that several genes have protective functions against aminoglycoside-induced HC loss. Overexpression of XIAP inhibits caspase expression and prevents neomycin-induced HC death and subsequent hearing loss.^25^ Insulin-like growth factor 1 (IGF-1) protects HCs from aminoglycosides by upregulating growth-associated protein 43 and netrin 1.^40^ In many organs and cell lines, Wnt/*β*-catenin has been reported to have a protective function against apoptosis. In human HCT116 colon cancer cells, Wnt/*β*-catenin negatively regulates the pro-apoptotic transcription factor Foxo3 and inhibits Foxo3-induced apoptosis.^28^ In the liver, Wnt/*β*-catenin protects against hepatotoxin DDC-induced liver injury and inhibits Foxo3 expression thus inhibiting oxidative stress-induced apoptosis.^14^ In the rat sensory epithelium OC1 cell line, Wnt/*β*-catenin protects the OC1 cells against cisplatin-induced cell death.^41^ Here, we found that after neomycin treatment overexpression of *β*-catenin in mouse HCs significantly inhibits the expression of Foxo3 and Bim (Figure 8), enhances the expression of antioxidant enzymes (Figure 9), reduces ROS accumulation (Figure 9), and inhibits caspase-induced apoptosis (Figures 6 and 7), and thus protects HCs against neomycin-induced damage (Figures 3 and 5). These results indicate that Wnt/*β*-catenin has an important role in protecting against neomycin-induced HC damage.

Aminoglycosides are widely used in clinics to treat bacterial infections, but all aminoglycosides have ototoxic side effects, which limit their clinical use. Mammalian sensory HCs have many mitochondria and high oxygen consumption, which makes them very sensitive to oxidative stress, especially when challenged by external stimulation such as noise or aminoglycosides.^42^ Here, we found that knockout of *β*-catenin in HCs increases caspase-mediated HC apoptosis after neomycin treatment and that overexpression of *β*-catenin in HCs inhibits caspase-mediated HC apoptosis after neomycin treatment (Figures 6 and 7). Besides the canonical Wnt/*β*-catenin pathway, two *β*-catenin-independent pathways have been described, including the Wnt/Ca^2+^ and Wnt/PCP (planar cell polarity) pathways.^43, 44^ In the zebrafish lateral line, after exposure to aminoglycosides, dying HCs undergo a transient increase in intracellular Ca^2+^ that occurs shortly after mitochondrial membrane potential collapse. Inhibition of intracellular Ca^2+^ elevation mitigates toxic effects of aminoglycoside exposure.^45^ Under physiological conditions, calcium and ROS act as signaling molecules inside the cell and their pathways can interact. However, under pathological conditions dysfunction in either of the systems might affect the other system thus potentiating harmful effects that might contribute to cell death.^46^ The role of non-canonical Wnt signaling in cochlear HC survival and the role of calcium overload in *β*-catenin deficiency-induced increased HC susceptibility to neomycin need to be investigated in the future.

Previous studies reported that Wnt/*β*-catenin is protective against oxidative stress-induced apoptosis through inhibition of Foxo3 in the liver, bones, and SH-SY5Y cells.^14, 15, 47^ There are also other papers that suggest that Wnt has the opposite effect on mitochondria. Yoon *et al.*^48^ reports that increased Wnt signals are a potent activator of mitochondrial biogenesis and ROS generation, leading to DNA damage and acceleration of cellular senescence in primary cells.^48^ This might be because Wnt signaling has diverse functions in different environments and stages, and sometimes these effects are in opposition to each other. For example, promoting proliferation and promoting differentiation are usually two opposing effects, and during embryonic stages Wnt/*β*-catenin signaling promotes proliferation during early mitotic phases of development and also promotes HC differentiation in the differentiating organ of Corti.^3^ Here, we found that neomycin-induced HC damage was accompanied by Foxo3 upregulation and mitochondrial ROS accumulation. Knockout of *β*-catenin in HCs upregulated Foxo3 expression and increased the accumulation of ROS even more, while overexpression of *β*-catenin in HCs inhibited Foxo3 expression and decreased the accumulation of ROS after neomycin injury. This finding indicates that Wnt/*β*-catenin protects HCs against neomycin injury by regulating Foxo3 expression and controlling ROS levels.

In summary, we showed that deletion of *β*-catenin in HCs increases neomycin-induced HC loss. Next, we reported that overexpression of *β*-catenin in HCs protects against neomycin-induced HC loss. Last, we demonstrated that Wnt/*β*-catenin signaling in HCs regulates Foxo3 expression, antioxidant enzymes, and ROS levels, thus protecting HCs against caspase-mediated apoptosis after neomycin injury. Our data suggest that Wnt/*β*-catenin signaling is essential for HC protection against neomycin-induced HC loss, and thus might be a new therapeutic target for the prevention of aminoglycoside-induced HC death.

## Materials and Methods

### Mouse models and treatments

We used C57BL/6 J WT mice and transgenic mice in the C57BL/6 J background. *β*-Catenin^flox(exon3)^ mice^49^ were generously provided by Mark Taketo (Kyoto University, Kyoto, Japan), *β*-catenin^flox(exon2–6)^ mice^50^ were ordered from The Jackson Laboratory (Bar Harbor, ME, USA; JAX number 004152), Rosa26-tdTomato mice^51^ were ordered from The Jackson Laboratory (JAX number 007914), and Gfi1-Cre mice^24^ were generously provided by Lin Gan (University of Rochester, Rochester, NY, USA). For *β*-catenin^flox(exon2–6)^ mice, Cre-induced recombination at the LoxP sites flanks exons 2–6 of *β*-catenin, and Cre activation results in a *β*-catenin-null allele.^50^ For *β*-catenin^flox(exon3)^ mice, *β-Catenin:exon3* encodes the GSK3 phosphorylation sites for degradation, and exon 3 is not functionally required for *β*-catenin transcriptional activity. Deletion of *β-catenin:exon3* therefore results in increased levels of *β*-catenin.

Mice were housed with open access to food and water at the Experimental Animal Center, Shanghai Medical College of Fudan University, China. Postnatal day (P) 0 was defined as the day of birth. Mice received a daily subcutaneous injection of neomycin (200 mg/kg) or sterile saline from P7 to P14. This study was carried out in strict accordance with the 'Guiding Directive for Humane treatment of Laboratory Animals' issued by the Chinese National Ministry of Science and Technology in September 2006. All experiments were approved by the Shanghai Medical Experimental Animal Administrative Committee (Permit Number: 2009-0082). All efforts were made to minimize suffering and reduce the number of animals used.

### Organotypic culture of neonatal mice cochlea

The mice were killed at P2, then the cochlear sensory epithelium was isolated and seeded intact on a glass coverslip coated with Cell-Tak (BD Biosciences, Franklin Lakes, NJ, USA).^52^ The explanted cochleae were treated with 1 mM neomycin (Sigma-Aldrich, St. Louis, MO, USA) and/or 5 *μ*M Bio (Sigma-Aldrich) or 20 mM NAC (Sigma-Aldrich). PBS was used as the vehicle control.

### ABR test

The hearing thresholds of the mice were examined with the ABR test. In this test, changes in the electrical activity of the brain in response to sound were recorded via electrodes that were placed on the scalp of the mice. Animals were anesthetized with ketamine (100 mg/kg) and xylazine (25 mg/kg) and placed on a thermostatic heating pad in a sound-attenuating chamber to maintain their body temperatures at 38 °C. Frequency-specific auditory responses were measured using the Tucker-Davis Technology system III (Tucker-Davies Technologies, Gainesville, FL, USA) as previously described.^53^ All ABR tests were performed on mice older than P21.

### Tissue preparation for quantitative RT-PCR and western blot

After killing the mice, the otic capsule was immediately isolated, rapidly frozen in liquid nitrogen, and stored at −70 °C until further processing. To obtain the total RNA, 10 cochleae were pooled in TRIzol (Invitrogen, Carlsbad, CA, USA) following the manufacturer's instructions. The RNA concentration was measured with a Bio-Rad spectrophotometer (Applied Biosystems, Foster City, CA, USA). cDNA was synthesized from 1 *μ*g total RNA by reverse transcription using the GoScript Reverse Transcription System (Promega, Madison, WI, USA) following the manufacturer's protocols. qPCR was performed using GoTaq qPCR Master Mix (Promega) on a Bio-Rad 7500 detection system (Applied Biosystems, Foster City, CA, USA). GAPDH was used as a housekeeping gene for control purposes. Primer sequences are listed in Supplementary Table 6. For protein extraction, 10 cochleae were pooled in 100 *μ*l RIPA lysis buffer with 1% PMSF, sonicated, incubated on ice for 30 min, and stored at −80 °C. Extracts were boiled with 5 × loading buffer, subjected to PAGE (Mini-Protean TGX Systems; Bio-Rad, Hercules, CA, USA), transferred onto an Immobilon-P membrane (Millipore, Bedford, MA, USA), probed with anti-Foxo3 antibody (Cell Signaling Technology, Danvers, MA, USA), anti-*β*-catenin (BD Biosciences), and anti-GAPDH (Kangchen Biotech, Shanghai, China) and finally incubated with HRP-conjugated secondary antibodies. Signal was detected with the Supersignal West Femto Trial Kit (Thermo Fisher Scientific, Rockford, IL, USA) on a FluorChem M system (ProteinSimple, San Jose, CA, USA).

### Immunofluorescence

After fixation, cochlear samples were blocked with 10% normal donkey serum in 10 mM phosphate-buffered saline (PBS, pH 7.4) with 0.3% Triton X-100 for 1 h at room temperature and then incubated with primary antibody overnight at 4 °C. The next day, the tissues were incubated for 2 h at 4 °C with 488- or 594-conjugated donkey secondary antibody (Invitrogen) and DAPI (Sigma-Aldrich). Omission of primary antibody served as the negative control. The following primary antibodies were used: anti-*β*-catenin (BD Biosciences), anti-myosin VIIA (Myosin7a) (Proteus Biosciences, Ramona, CA, USA), anti-cleaved caspase-3 (Cell Signaling Technology), anti-Foxo3 (Cell Signaling Technology), anti-Parvalbumin (Sigma-Aldrich), TUNEL (Roche, Indianapolis, IN, USA), and MitoSOX Red (Life Technologies, Rockford, IL, USA). Cochleae were dissected into apical, middle, and basal turns, and images were taken using a Leica SP5 confocal fluorescence microscope (Leica Microsystems, Biberach, Germany).

### Fluorescence activated cell sorting

Gfi1-Cre/*β*-catenin^flox(exon2–6)^/Rosa26-tdTomato or Gfi1-Cre/*β*-catenin^flox(exon3)^/Rosa26-tdTomato mice (P2) were dissected out to isolate ∼20–40 cochleae, and then trypsinized with prewarmed 0.125% trypsin/EDTA (Invitrogen) at 37 °C for 8 min. Soybean trypsin inhibitor (Worthington Biochem, Lakewood, CO, USA) was added to terminate the reaction followed by mechanical trituration with blunt tips and pipetting up and down up to 80–100 times. Suspended cells were percolated through a 40-μm cell strainer before fluorescence activated cell sorting (FACS). tdTomato-positive HCs were sorted out on a BD FACS Aria III (BD Biosciences) using the tdTomato channel.

### Cell counts

For HC quantification in the neomycin-treated samples, we imaged the entire cochlea using a 40 × 3 objective and counted the Myosin7a+ HCs that remained. The same procedure was used to quantify cleaved caspase-3+/Myosin7a+, TUNEL+/Myosin7a+ cells, and myosin7a+/MitoSOX Red+ cells. For all experiments, only one cochlea from each mouse was used for immunofluorescence and quantification. Thus, *n* represents the number of mice examined.

### Statistical analyses

Statistical analyses were conducted using Microsoft Excel and GraphPad Prism software (GraphPad Software, La Jolla, CA, USA). Data were expressed as mean±S.E.M. ABR thresholds were analyzed by two-way ANOVA followed by a Newman–Keuls *post hoc* test. Immunofluorescence analysis was performed with a two-tailed, unpaired Student's *t*-test when comparing two groups or with a one-way ANOVA followed by a Dunnett's multiple comparisons test when comparing more than two groups. *P*<0.05 was considered as statistically significant.

## Abbreviations

ABR: auditory brainstem response
Bio: (2'Z,3'E)-6-Bromoindirubin-3'-oxime
HC: hair cell
OHC: outer hair cell
IHC: inner hair cell
ROS: reactive oxygen species
P: postnatal day
PFA: paraformaldehyde
Foxo3: forkhead box O3 transcription factor
Foxo1: forkhead box O1 transcription factor
Sgk1: glucocorticoid-inducible kinase
qPCR: quantitative real-time PCR
DAPI: 4,6-diamidino-2-phenylindole
Myosin7a: MyosinVIIA
TUNEL: transferase-mediated deoxyuridine triphosphate-biotin nick end labeling
NAC: antioxidant *N*-acetylcysteine
Nqo1: NAD(P)H dehydrogenase, quinone 1
Sod1: superoxide dismutase 1
Sod2: superoxide dismutase 2
Gsr: glutathione reductase
Cat: catalase
PMSF: phenylmethanesulphonyl fluoride
BIM: Bcl-2-like protein 11
FM1-43: N-(3-Triethylammoniumpropyl)-4-(4-(Dibutylamino) Styryl) Pyridinium Dibromide
