# Supplementary figures and images for: Wnt activation protects against neomycin-induced hair cell damage in the mouse cochlea
Source: Cell Death Dis. 2016 Mar 10;7(3):e2136–. doi: 10.1038/cddis.2016.35 (PMC4823936; doi:10.1038/cddis.2016.35)

Supplemental tables:


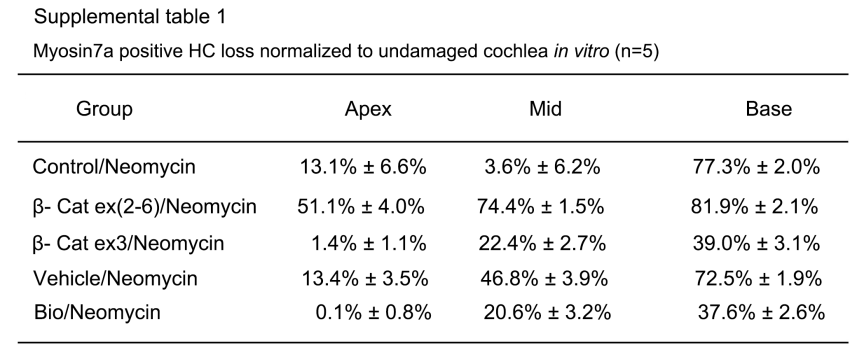


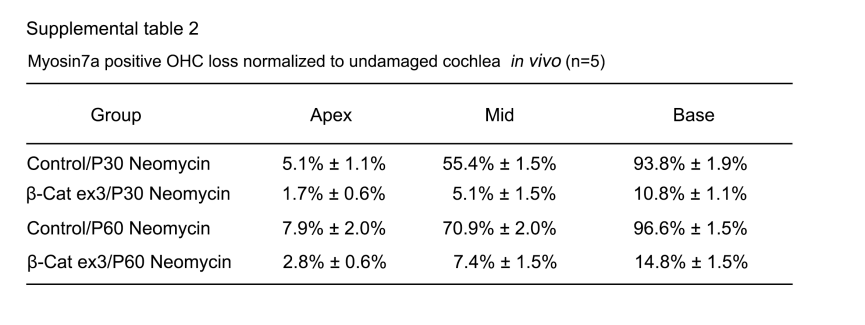


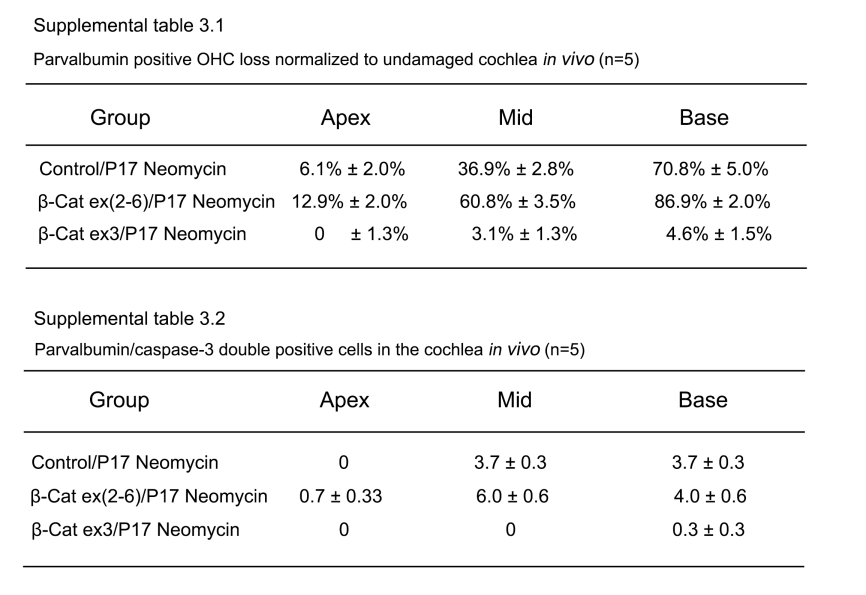


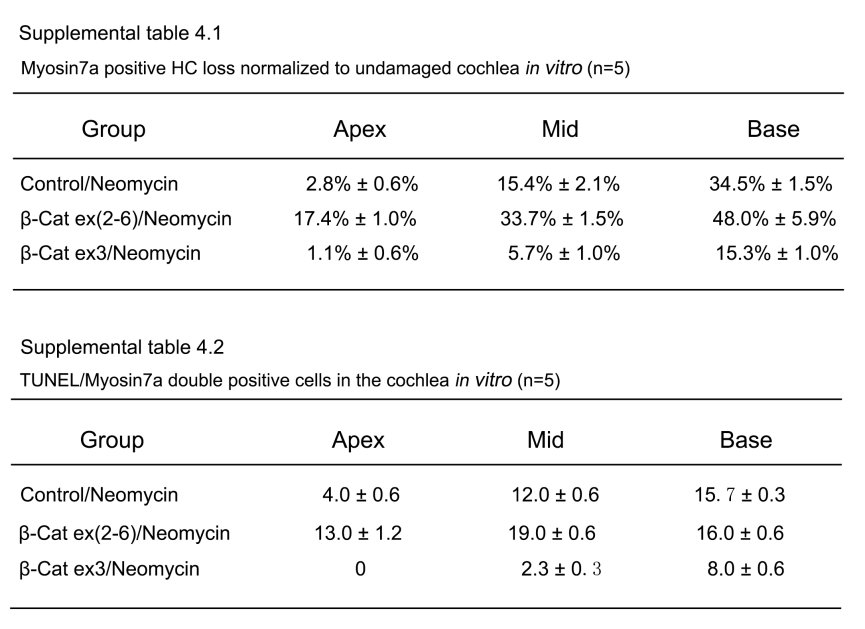


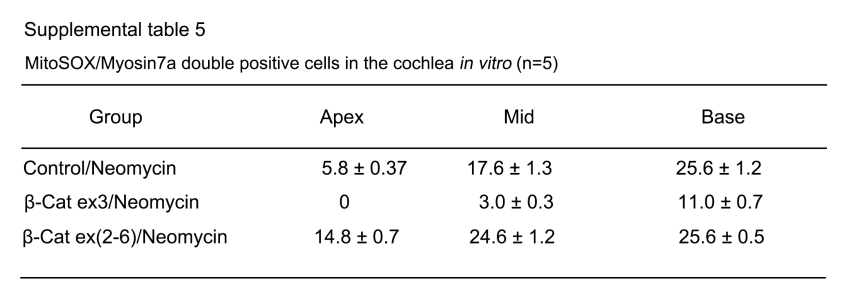


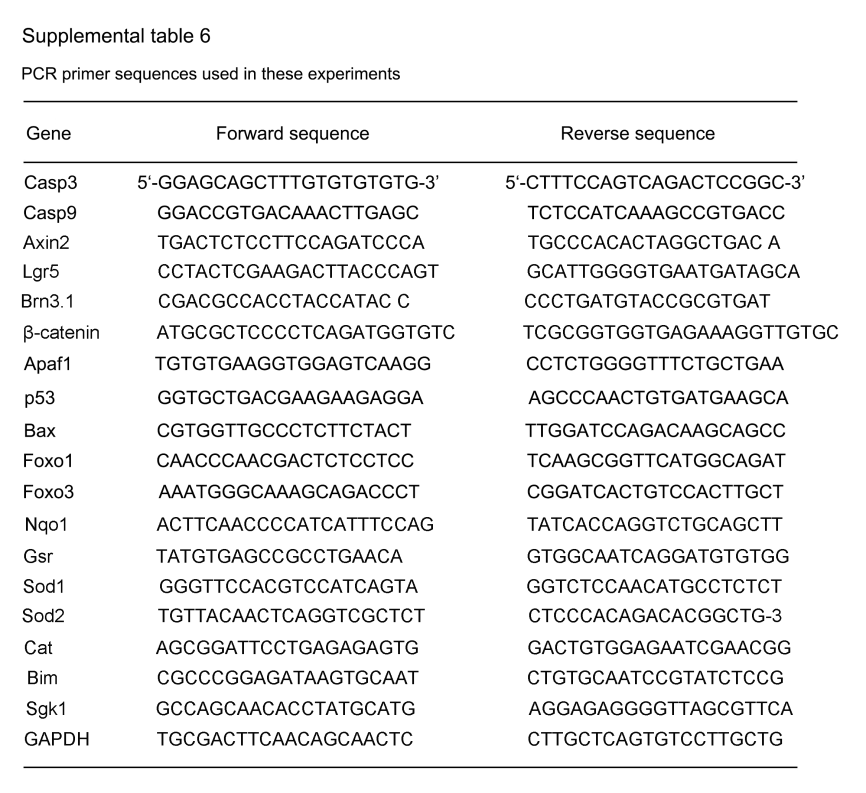

Supplement: Supplementary Tables [file cddis201635x1.doc]
